# Copper (Cu^2+^) Inhibits Voltage-Dependent Ionic Currents While Enhancing Neurotransmitter Release in Bovine Chromaffin Cells

**DOI:** 10.3390/ph19050716

**Published:** 2026-04-30

**Authors:** Víctor Varea-Tierno, Victoria Jiménez Carretero, Minerva Reyes Almodóvar, Javier Hernández Campano, María Arribas Tejedor, Ricardo de Pascual, Jesús M. Hernández-Guijo

**Affiliations:** 1 Department of Pharmacology and Therapeutics, Facultad de Medicina, Universidad Autónoma de Madrid, Av. Arzobispo Morcillo 4, 28029 Madrid, Spain; victor.varea@estudiante.uam.es (V.V.-T.); victoria.jimenez@uam.es (V.J.C.); minerva.reyes@uam.es (M.R.A.); esculebre@gmail.com (J.H.C.); mariaarribas2000@gmail.com (M.A.T.); ricardo.pascual@uam.es (R.d.P.); 2 Ramón y Cajal Institute for Health Research, IRYCIS, Hospital Ramón y Cajal, Ctra. de Colmenar Viejo, 28034 Madrid, Spain

**Keywords:** copper, chromaffin cells, patch-clamp, calcium currents, neurotransmitter release, cellular excitability, calcium channels, sodium channels

## Abstract

**Background/Objectives**: Copper (Cu^2+^) is an essential trace element that participates as a cofactor in key metabolic enzymes such as cytochrome c oxidase and superoxide dismutase. However, excessive copper exposure can be toxic and disturbances in copper homeostasis have been associated with neurodegenerative diseases including Alzheimer’s and Parkinson’s disease. Despite growing evidence linking copper to neuronal dysfunction, the cellular mechanisms by which Cu^2+^ affects neuronal excitability and neurotransmission remain poorly understood. The aim of this study was to investigate the effects of acute Cu^2+^ exposure on ionic currents involved in cellular excitability and neurotransmitter release in bovine chromaffin cells. **Methods**: Primary cultures of bovine chromaffin cells were used as a neuroendocrine model to study cellular excitability. Voltage-dependent ionic currents were recorded using the whole-cell patch-clamp technique in voltage-clamp configuration. Catecholamine secretion was monitored by amperometry, and cytosolic Ca^2+^ dynamics were measured in fluo-4-loaded cells during depolarization induced by high K^+^ stimulation. **Results**: Acute Cu^2+^ exposure produced a concentration-dependent enhancement of depolarization-evoked catecholamine release. In parallel, Cu^2+^ inhibited voltage-dependent calcium (ICa), sodium (INa), potassium (IKv), and calcium/voltage-dependent potassium (IKCa-v) currents in a concentration-dependent and partially reversible manner. In addition, Cu^2+^ increased basal cytosolic Ca^2+^ levels while reducing the amplitude of depolarization-evoked Ca^2+^ transients. **Conclusions**: Acute Cu^2+^ exposure exerts a dual effect in bovine chromaffin cells, inhibiting the ionic currents that support cellular excitability while potentiating catecholamine secretion. This apparent paradox is consistent with a disruption of intracellular Ca^2+^ homeostasis, in which elevated basal cytosolic Ca^2+^ may facilitate exocytosis despite reduced depolarization-evoked Ca^2+^ entry. These findings provide new insight into the mechanisms by which copper may alter neuronal signaling and contribute to neurotoxicity.

## 1. Introduction

Copper (Cu^2+^) is an essential trace element required for multiple biological processes. As a redox-active metal, copper acts as a cofactor for key enzymes involved in oxidative metabolism, antioxidant defense, iron homeostasis, and neurotransmitter synthesis, including cytochrome c oxidase, superoxide dismutase, ceruloplasmin, and dopamine-β-monooxygenase [1,2,3,4,5,6]. In the nervous system, copper also contributes to brain development, neuromodulation, and myelination [1,7,8,9,10]. However, because of its high redox reactivity, dysregulated copper can also promote oxidative stress and cellular damage [11].

In the brain, copper is tightly regulated and unevenly distributed among regions and subcellular compartments [10,12,13,14,15,16,17,18]. Although cerebrospinal fluid copper concentrations are relatively low, extracellular copper levels in neural tissue may rise substantially under physiological conditions, particularly after neuronal depolarization [19,20]. In addition, most brain copper is protein-bound and localized in cytosolic, mitochondrial, synaptic vesicle, synaptosomal, and endosomal compartments, supporting the idea that copper participates in neuronal signaling as well as in general cellular metabolism [10,15,18].

Increasing evidence indicates that copper modulates synaptic function. Copper has been reported to affect neurotransmission through actions on GABA and NMDA receptors and on voltage-gated ion channels, including Ca^2+^ and K^+^ channels [21,22,23,24,25,26,27]. These observations suggest that copper may influence neuronal excitability and stimulus–secretion coupling. At the same time, disruption of copper homeostasis has been linked to several neurological disorders, including Menkes disease, Wilson disease, Alzheimer’s disease, and Parkinson’s disease [1,15,28,29,30,31]. In these conditions, abnormal copper handling may contribute to neuronal dysfunction through oxidative stress, altered protein aggregation, and defective synaptic signaling.

Despite this growing evidence, the acute cellular mechanisms by which Cu^2+^ modifies excitability and neurotransmitter release are still not fully understood. In particular, it remains unclear how copper simultaneously affects the voltage-dependent ionic currents that support action potential generation and the calcium-dependent processes that govern exocytosis. A better definition of these mechanisms is important for understanding how copper excess may alter neuronal communication and contribute to neurotoxicity.

To address this issue, we used primary cultures of bovine chromaffin cells, a well-established neuroendocrine model for the study of cellular excitability and regulated catecholamine secretion [32,33,34]. Chromaffin cells share key functional properties with sympathetic neurons, including the expression of voltage-dependent Na^+^, Ca^2+^, and K^+^ channels and the capacity to synthesize, store, and release catecholamines [32,33,34]. Using this model, we investigated the acute effects of Cu^2+^ on catecholamine release, voltage-dependent Ca^2+^, Na^+^, K^+^, and Ca^2+^/voltage-dependent K^+^ currents, as well as on cytosolic Ca^2+^ dynamics. Our aim was to determine how copper alters the ionic and calcium-dependent mechanisms that control neurotransmitter release.

## 2. Results

### 2.1. Cu^2+^ Increases Neurotransmitter Release

Chromaffin cells constitute a well-established experimental model for the study of the molecular mechanisms underlying cellular excitability and regulated neurotransmitter secretion [35,36,37]. These cells express voltage-gated Na^+^, K^+^, and Ca^2+^ channels, which are essential for the maintenance of the resting membrane potential, the generation of action potentials, and the control of stimulus–secretion coupling [38].

**Figure 1:**
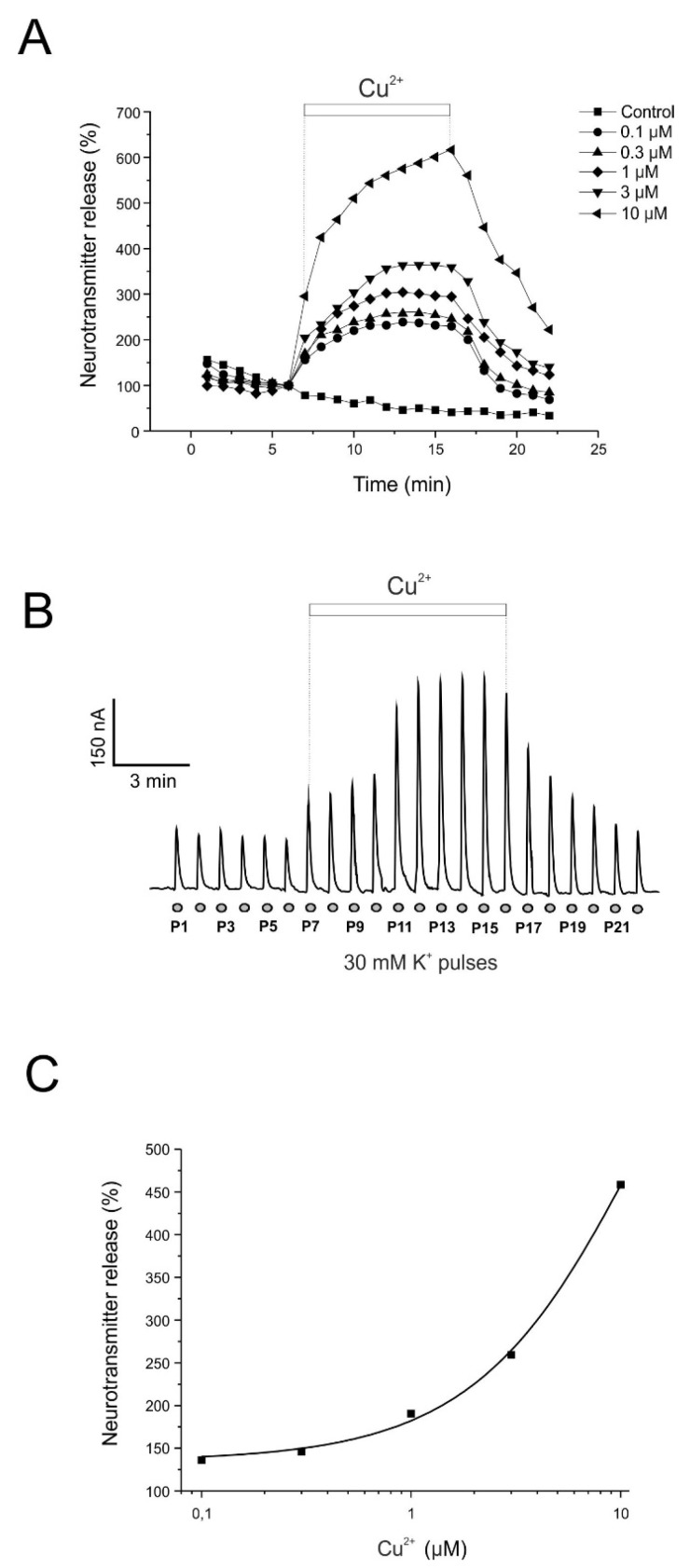
Cu^2+^ potentiates the catecholamine release responses obtained in fast-superfused cells triggered by K^+^ stimulation. Cells were superfused with Krebs–HEPES solution containing 2 mM Ca^2+^ and stimulated at 1 min intervals with 10 s pulses of 35 mM K^+^. Cu^2+^ was applied during the periods indicated by the horizontal bars. (**A**) Averaged time course of catecholamine secretion under control conditions and during superfusion with the indicated Cu^2+^ concentrations (shown by the horizontal bars). Each Cu^2+^ concentration was tested in a separate group of cells. (**B**) Representative amperometric recordings obtained in control cells and during superfusion with Cu^2+^ (3 μM), as indicated by the horizontal bar, indicated as P1 to P22. (**C**) Concentration–response curve showing the effects of Cu^2+^ on catecholamine release. Data points represent the percentage of neurotransmitter release potentiation (ordinate axis) obtained at different Cu^2+^ concentrations (abscissa axis) relative to control conditions. A separate cell population was used for each concentration. The data were fitted using a nonlinear regression function (*y* = 674.73 − 539.55 × 0.91*^x^*) to the averaged values of catecholamine release potentiation measured at pulse P16 after Cu^2+^ administration.

In the experiment shown in Figure 1, chromaffin cells superfused with Krebs–HEPES solution exhibited a stable basal spontaneous catecholamine release of approximately 2 nA. Depolarizing stimuli were applied using 10 s pulses of a solution containing 35 mM K^+^ (35K), which evoked catecholamine secretion peaks of approximately 197 nA. When K^+^ pulses were applied repeatedly at 1 min intervals, a gradual decline in secretion amplitude was observed, as shown in the control condition (Figure 1A).

To evaluate the effect of copper, cells were superfused with a single concentration of Cu^2+^ (0.1, 0.3, 1, 3, or 10 μM) from pulses P7 to P16. The presence of Cu^2+^ produced a marked increase in the amplitude of K^+^-evoked secretory responses. Figure 1A illustrates the normalized time course of catecholamine release in the presence of the different Cu^2+^ concentrations. Catecholamine secretion increased to 236%, 246%, 294%, 359%, and 559% for 0.1, 0.3, 1, 3, and 10 μM Cu^2+^, respectively, relative to pulse P6 (immediately before Cu^2+^ application), after correction for the spontaneous signal decay observed in control experiments. Following Cu^2+^ washout (pulse P17), secretion decreased markedly and returned to control levels.

Figure 1C shows the concentration–response relationship for Cu^2+^-induced potentiation of catecholamine release. At the end of Cu^2+^ application, catecholamine release potentiation reached 136.18 ± 13.10%, 146.04 ± 14.24%, 194.46 ± 22.29%, 259.26 ± 17.85%, and 458.54 ± 41.66% for 0.1, 0.3, 1, 3, and 10 μM Cu^2+^, respectively, relative to pulse P6, taking into account the 31.12% spontaneous signal reduction observed in control experiments at pulse P16.

### 2.2. Time- and Concentration-Dependent Blockade of ICa by Copper

**Figure 2:**
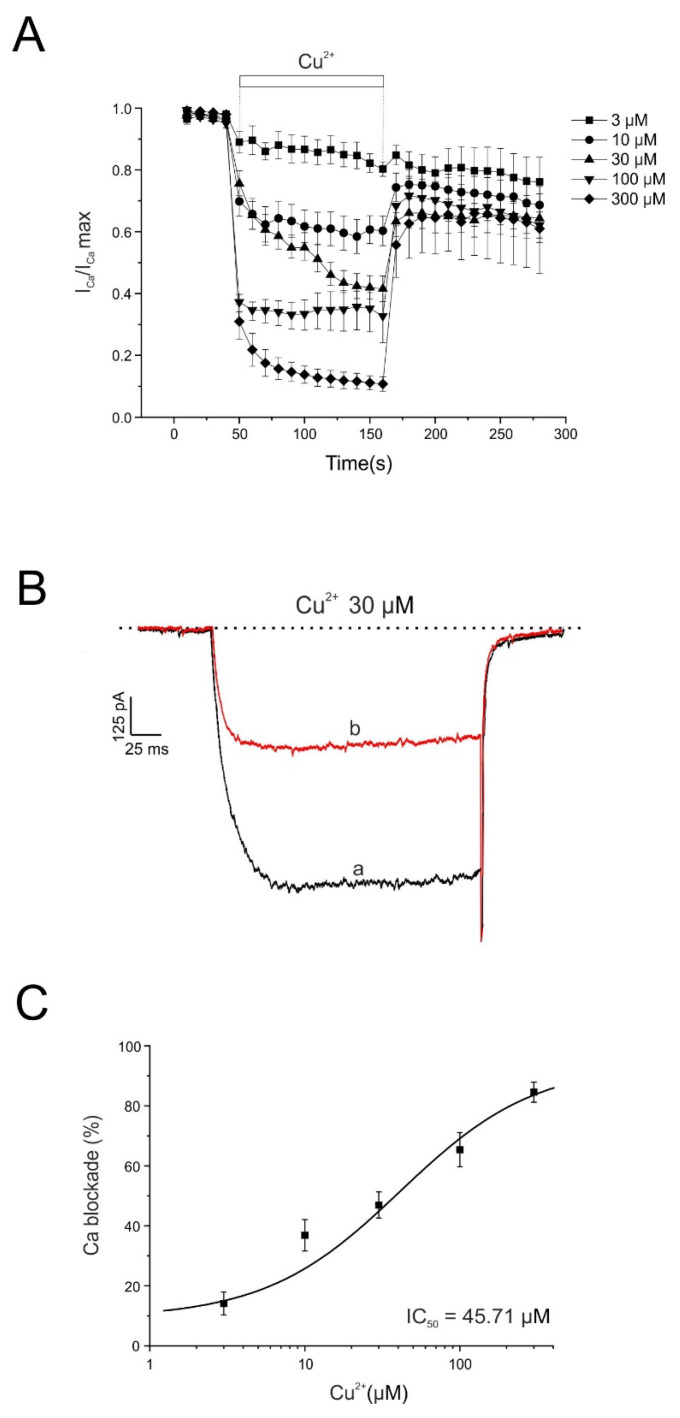
Time course of the inhibition by Cu^2+^ of the whole-cell inward Ca^2+^ current. (**A**) Averaged time course of I_Ca under control conditions and during superfusion with different Cu^2+^ concentrations applied during the periods indicated by the horizontal bars. Tetrodotoxin (TTX, 1 µM) was included to prevent activation of Na^+^ currents. Horizontal bars indicate the superfusion periods for the Cu^2+^ concentrations shown on the right. A different cell was used for each Cu^2+^ concentration. (**B**) Representative current traces recorded under control conditions (a) and at the end of superfusion with Cu^2+^ (30 µM) (b). (**C**) Concentration–response relationship for Cu^2+^-induced inhibition of I_Ca_. Data represent the percentage of current inhibition (ordinate axis) after 2 min superfusion with each Cu^2+^ concentration (abscissa axis). The average data were fitted using a sigmoidal Hill equation: y = (1.10 · x^0.63^)/(45.71^0.63^ + x^0.63^). The calculated IC_50_ value was 45.71 µM. Data were normalized to the mean control value and are expressed as mean ± SEM (n = 7 experiments; Wilcoxon matched-pairs signed-rank test).

In a second series of experiments, we investigated whether the modulation of calcium conductance involved in neurotransmitter secretion could account for the Cu^2+^-induced increase in catecholamine release. In the experiments shown in Figure 2, individual voltage-clamped chromaffin cells were stimulated with 50 ms depolarizing pulses to 0 mV, applied at 10 s intervals from a holding potential of −80 mV. Inward calcium currents (I_Ca_) were recorded using 10 mM extracellular Ca^2+^ as the charge carrier. In 67 cells tested, the mean peak current amplitude was 685 ± 50 pA. This current remained stable during the approximately 5 min recording period; cells showing noticeable current rundown were excluded from the analysis.

Once the current reached a stable baseline, each cell was superfused with a single concentration of Cu^2+^ until the effect stabilized for at least 2 min. Partial washout of Cu^2+^ prevented the construction of cumulative concentration–response curves in the same cell. To minimize variability between recordings, currents were normalized to the maximal current obtained at the beginning of each experiment (I_Ca_/I_Ca_ max).

Figure 2A shows the averaged time course of I_Ca_ inhibition produced by five Cu^2+^ concentrations (3, 10, 30, 100, and 300 µM), demonstrating a clear concentration-dependent blocking effect. Figure 2B shows representative current traces under control conditions and after 2 min superfusion with Cu^2+^ (30 μM). Figure 2C illustrates the concentration–response curve for Cu^2+^-induced inhibition of peak I_Ca_. The degree of inhibition measured at the end of the superfusion period was 14 ± 4%, 37 ± 5%, 47 ± 4%, 65 ± 6%, and 85 ± 3% for 3, 10, 30, 100, and 300 μM Cu^2+^, respectively (n = 7). The IC_50_ value was 45.7 μM. After washout, partial recovery of the current was observed, reaching 76 ± 8%, 69 ± 6%, 64 ± 8%, 62 ± 4%, and 61 ± 9% of the control current for 3, 10, 30, 100, and 300 μM Cu^2+^, respectively.

**Figure 3:**
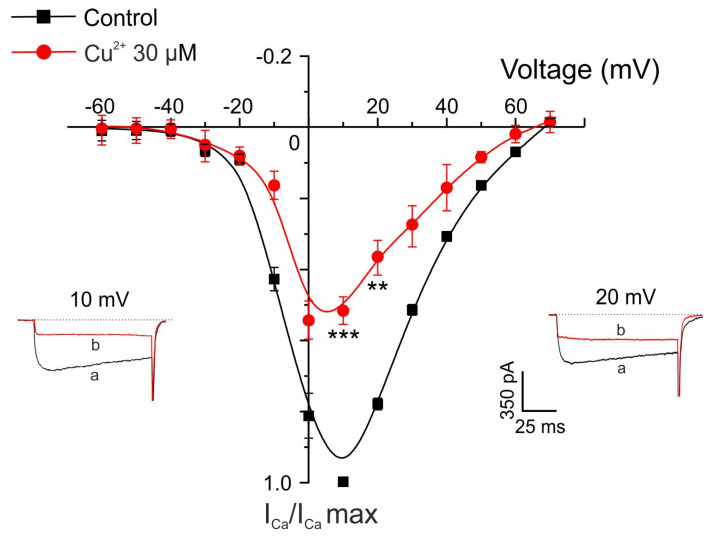
Voltage/Ca^2+^-current relationship obtained before and after perfusing with Cu^2+^. Test depolarizing pulses were applied at the indicated voltages (abscissa axis), and the averaged current amplitude is plotted on the ordinate axis under control conditions (black trace) and after 2 min superfusion with 30 µM Cu^2+^ (red trace). Insets show representative current traces recorded at different test potentials under control conditions (a) and after 2 min Cu^2+^ superfusion (b). For each cell, data were normalized to the maximum current recorded under control conditions and are expressed as mean ± SEM (n = 5 experiments). ** *p* < 0.01 and *** *p* < 0.001 compared with control (Wilcoxon matched-pairs signed-rank test).

In an additional set of experiments, voltage-clamped chromaffin cells were stimulated with depolarizing pulses (50 ms) of increasing amplitude applied at 10 s intervals from a holding potential of −80 mV, before and after 2 min superfusion with Cu^2+^, using 10 mM Ca^2+^ as the charge carrier. The current–voltage (I–V) relationship under control conditions showed that peak I_Ca_ activation began at approximately −20 mV, reached a maximum at +10 mV, and reversed near +60 mV (Figure 3). After 2 min exposure to Cu^2+^ (30 μM), the I–V relationship showed a slight shift toward more negative potentials, with activation beginning around −30 mV and the peak current occurring at 0 mV. Additionally, a statistically significant reduction in I_Ca_ amplitude was observed at 10 and 20 mV (see original traces in Figure 3).

### 2.3. Time- and Concentration-Dependent Blockade of I_Na_ by Copper

**Figure 4:**
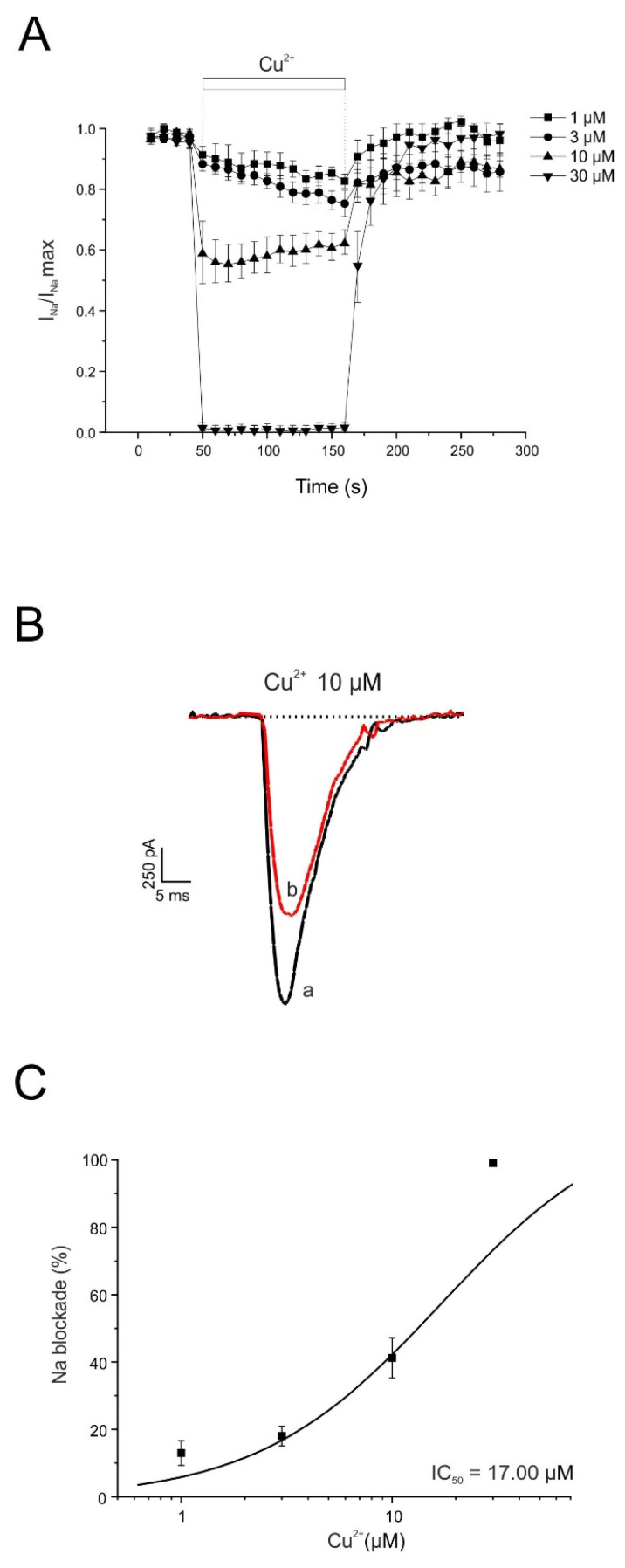
Time course of the inhibition by Cu^2+^ of the whole-cell inward Na^+^ current. (**A**) Averaged time course of I_Na_ under control conditions and during superfusion with the indicated Cu^2+^ concentrations applied during the periods shown by the horizontal bars. Horizontal bars indicate the superfusion periods for the Cu^2+^ concentrations shown on the right. A different cell was used for each Cu^2+^ concentration. (**B**) Representative current traces recorded under control conditions (a) and at the end of superfusion with Cu^2+^ (10 µM) (b). (**C**) Concentration–response relationship for Cu^2+^-induced inhibition of I_Na_. Data represent the percentage of current inhibition (ordinate axis) measured after 2 min superfusion with each Cu^2+^ concentration (abscissa axis). The averaged data were fitted using a sigmoidal Hill equation: y = (0.99 · x)/(17.00 + x).

In a subsequent series of experiments, we evaluated the effect of Cu^2+^ on voltage-gated sodium channels, which are responsible for the initiation and propagation of action potentials. In the experiments shown in Figure 4, individual voltage-clamped chromaffin cells were stimulated with 10 ms depolarizing pulses to −10 mV, applied at 10 s intervals from a holding potential of −80 mV. The mean initial sodium current (I_Na_) amplitude was 406 ± 45 pA (n = 27). The current remained stable during the approximately 5 min recording period; cells showing signs of current rundown were excluded from analysis.

After stabilization of the initial current, each cell was superfused with a single concentration of Cu^2+^ until the effect reached a steady state for at least 2 min. To reduce variability between recordings, currents were normalized to the maximal current measured at the beginning of each experiment (I_Na_/I_Na_ max).

Figure 4A shows the averaged time course of I_Na_ inhibition produced by four concentrations of Cu^2+^ (1, 3, 10, and 30 μM), demonstrating a clear concentration-dependent blocking effect. Figure 4B presents representative current traces under control conditions and after 2 min superfusion with Cu^2+^ (10 μM). Figure 4C shows the concentration–response relationship for Cu^2+^-induced inhibition of peak I_Na_. The inhibition measured at the end of the 2 min superfusion period was 13 ± 4%, 18 ± 3%, 41 ± 6%, and 99 ± 0.7% for 1, 3, 10, and 30 μM Cu^2+^, respectively (n = 5–6). The calculated IC_50_ value for Cu^2+^ inhibition of I_Na_ was 17.00 μM.

The calculated IC_50_ value was 17.00 µM. Data were normalized to the mean control value and are expressed as mean ± SEM (n = 5–10 experiments; Wilcoxon matched-pairs signed-rank test).

**Figure 5:**
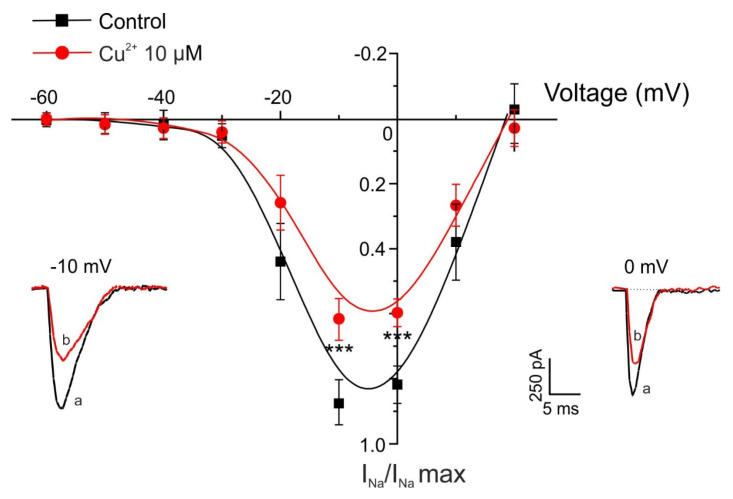
Voltage/Na^+^-current relationship obtained before and after perfusing with Cu^2+^. Test depolarizing pulses were applied at the indicated voltages (abscissa axis), and the averaged current amplitude is plotted on the ordinate axis under control conditions (black trace) and after 2 min superfusion with 10 µM Cu^2+^ (red trace). Insets show representative current traces recorded at different test potentials under control conditions (a) and after 2 min Cu^2+^ superfusion (b). For each cell, data were normalized to the maximum current recorded under control conditions and are expressed as mean ± SEM (n = 5 cells). *** *p* < 0.001 compared with control (Wilcoxon matched-pairs signed-rank test).

A separate set of voltage-clamped chromaffin cells was stimulated with 10 ms depolarizing pulses of increasing amplitude applied at 10 s intervals from a holding potential of −80 mV, both before and 2 min after superfusion with Cu^2+^. The current–voltage (I–V) relationship under control conditions showed that peak I_Na activation began at approximately −30 mV, reached a maximum at −10 mV, and reversed near +20 mV (Figure 5). After 2 min exposure to Cu^2+^ (10 μM), no significant shift in the I–V relationship was observed. However, Cu^2+^ produced a stronger inhibition of I_Na at more negative potentials than at positive potentials, as illustrated in the original current traces (Figure 5).

### 2.4. Time- and Concentration-Dependent Blockade of I_Kv_ by Copper

**Figure 6:**
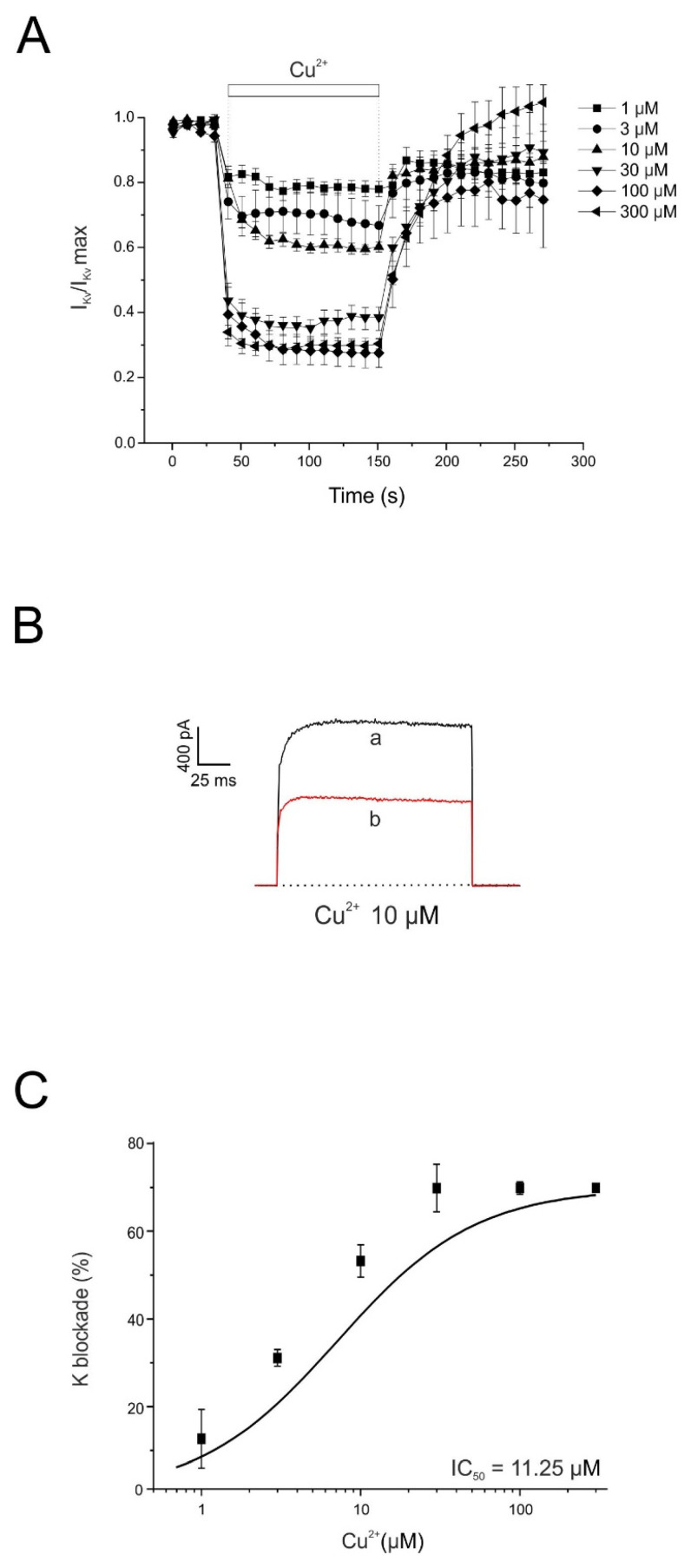
Time course of the inhibition by Cu^2+^ of the whole-cell outward K^+^ current. (**A**) Averaged time course of I_Kv under control conditions and during superfusion with the indicated Cu^2+^ concentrations applied during the periods shown by the horizontal bars. Horizontal bars indicate the superfusion periods for the Cu^2+^ concentrations shown on the right. A different cell was used for each Cu^2+^ concentration. (**B**) Representative current traces recorded under control conditions (a) and at the end of superfusion with Cu^2+^ (10 µM) (b). (**C**) Concentration–response relationship for Cu^2+^-induced inhibition of I_Kv. Data represent the percentage of current inhibition (ordinate axis) measured after 2 min superfusion with each Cu^2+^ concentration (abscissa axis). The averaged data were fitted using a sigmoidal Hill equation: y = (0.83 · x^0.52)/(11.25^0.52 + x^0.52). The calculated IC_50_ value was 11.25 µM. Data were normalized to the mean control value and are expressed as mean ± SEM (n = 5–7 experiments; Wilcoxon matched-pairs signed-rank test).

In chromaffin cells, as in most excitable cell types, K^+^ currents are primarily responsible for membrane repolarization. In these cells, potassium currents are mediated by both voltage-dependent K^+^ channels and Ca^2+^-activated voltage-dependent K^+^ channels [39]. In the experiments shown in Figure 6, the effects of Cu^2+^ on the voltage-dependent potassium current (I_Kv_) were investigated.

Individual voltage-clamped cells were stimulated with 45 ms depolarizing pulses to +120 mV, applied at 10 s intervals from a holding potential of −80 mV. In 48 cells tested, the mean current amplitude was 1637 ± 134 pA. The current remained stable during the approximately 5 min recording period, and cells showing signs of current rundown were excluded from the analysis.

Once the initial current stabilized, each cell was superfused with a single concentration of Cu^2+^ until the effect reached a steady state after approximately 2 min. Partial washout of Cu^2+^ prevented the construction of cumulative concentration–response curves in the same cell. To minimize variability between recordings, currents were normalized to the maximal current recorded at the beginning of each experiment (I_Kv_/I_Kv_ max).

Figure 6A shows the averaged time course of I_Kv inhibition produced by six concentrations of Cu^2+^ (1, 3, 10, 30, 100, and 300 μM), demonstrating a concentration-dependent inhibitory effect. Figure 6B shows representative current traces under control conditions and after 2 min superfusion with Cu^2+^ (10 μM). Figure 6C shows the concentration–response relationship for Cu^2+^-induced inhibition of peak I_Kv_. The inhibition measured at the end of the 2 min superfusion period was 21 ± 2%, 30 ± 6%, 37 ± 2%, 62 ± 4%, 70 ± 5%, and 70 ± 1% for 1, 3, 10, 30, 100, and 300 μM Cu^2+^, respectively (n = 5–7). The calculated IC_50_ value for Cu^2+^ inhibition of I_Kv was 11.25 μM.

**Figure 7:**
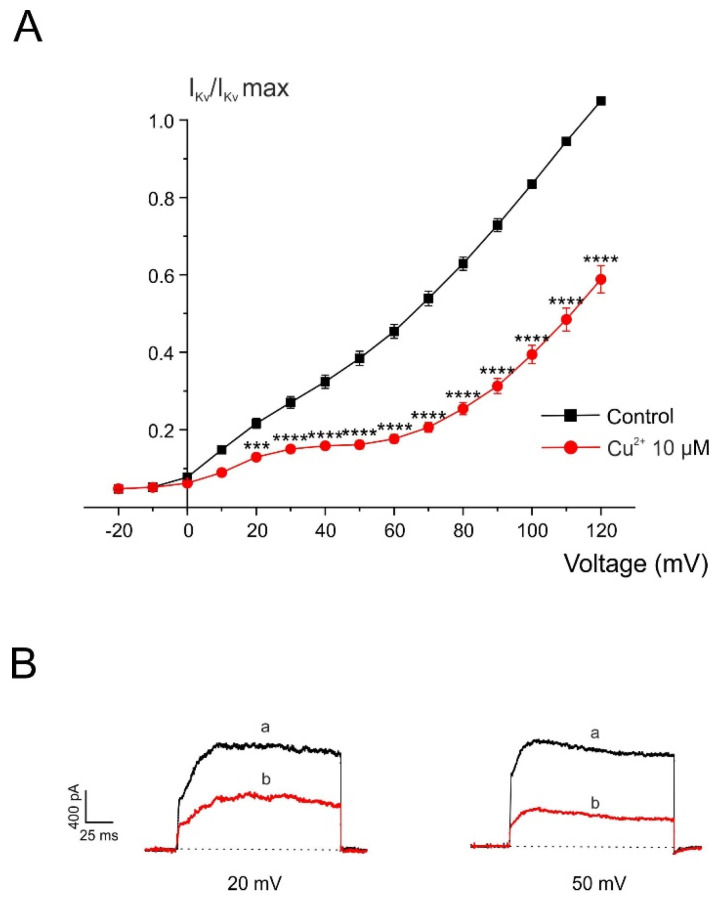
Voltage/Kv-current relationship obtained before and after perfusing with Cu^2+^. (**A**) Test depolarizing pulses were applied at the indicated voltages (abscissa axis), and the averaged current amplitude is plotted on the ordinate axis under control conditions (black trace) and after 2 min superfusion with 10 µM Cu^2+^ (red trace). (**B**) Representative current traces recorded at different test potentials under control conditions (a) and after 2 min Cu^2+^ superfusion (b). For each cell, data were normalized to the maximum current recorded under control conditions and are expressed as mean ± SEM (n = 5 experiments). *** *p* < 0.001 and **** *p* < 0.0001 compared with control (Wilcoxon matched-pairs signed-rank test).

A separate set of voltage-clamped chromaffin cells was stimulated with 45 ms depolarizing pulses of increasing amplitude applied at 10 s intervals from a holding potential of −80 mV, both before and after 2 min superfusion with Cu^2+^. The current–voltage (I–V) relationship under control conditions showed that I_Kv activation began at approximately −25 mV (Figure 7). After 2 min exposure to Cu^2+^ (10 μM), the I–V relationship did not show significant changes in its kinetic parameters. However, Cu^2+^ reduced the current amplitude at potentials of +20 mV and above, indicating a decrease in current magnitude without significant alterations in activation properties.

### 2.5. Copper Induced a Blockade of the Calcium Modulated Voltage-Dependent Potassium Channels

Activation of outward K^+^ currents is essential for the rapid repolarization and termination of action potentials in chromaffin cells. Although the precise spatial distribution of Ca^2+^ channels in chromaffin cells remains unclear, it has been suggested that voltage-dependent Ca^2+^ channels are located in close proximity to BK channels [40,41].

**Figure 8:**
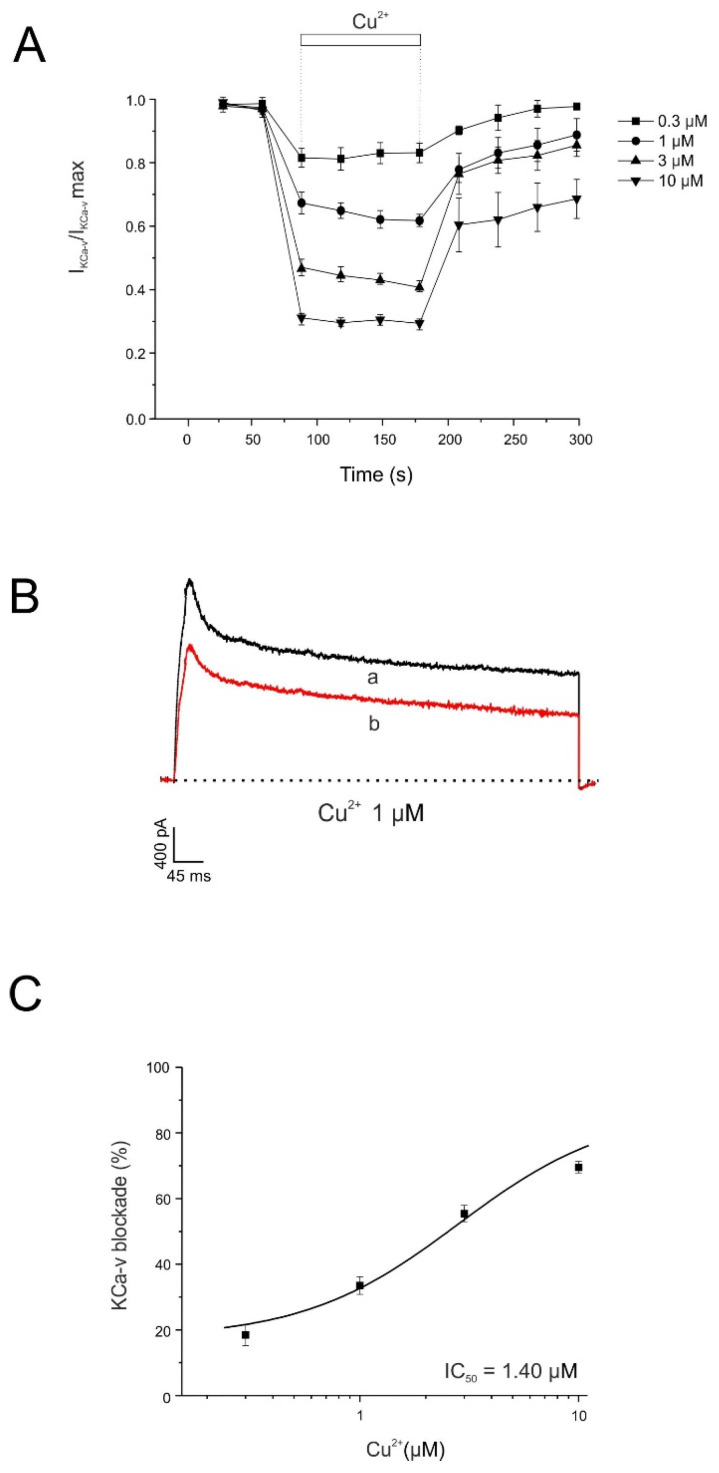
Cu^2+^ depresses BK channel activation in a concentration dependent manner. (**A**) Averaged time course of I_KCa-v obtained using the protocol shown in panel B, under control conditions and during superfusion with the indicated Cu^2+^ concentrations applied during the periods shown by the horizontal bars. (**B**) Representative current traces recorded under control conditions (a) and at the end of superfusion with Cu^2+^ (1 µM) (b). (**C**) Concentration–response relationship for Cu^2+^-induced inhibition of I_KCa-v_. Data represent the percentage of current inhibition (ordinate axis) measured after 2 min superfusion with each Cu^2+^ concentration (abscissa axis). The averaged data were fitted using a sigmoidal Hill equation: y = (0.55 · x)/(1.39 + x). The calculated IC_50_ value was 1.39 µM. Data were normalized to the mean control value and are expressed as mean ± SEM (n = 7 experiments; Wilcoxon matched-pairs signed-rank test).

In the experiments shown in Figure 8, cells were first depolarized to +20 mV for 30 ms to induce Ca^2+^ influx through voltage-dependent Ca^2+^ channels. This pre-pulse was followed by a depolarizing step to +120 mV for 400 ms from a holding potential of −80 mV. At +120 mV, Ca^2+^ influx ceases, and the Ca^2+^-activated voltage-dependent potassium current (I_KCa-v_) is activated. In the absence of the 30 ms pre-pulse, no Ca^2+^-dependent current was activated, and the outward current recorded corresponded exclusively to Ca^2+^-independent voltage-activated K^+^ channels. Following the Ca^2+^ influx induced by the pre-pulse, more than 90% of the total K^+^ current became available for activation. The I_KCa-v_ current decayed rapidly and completely after closure of Ca^2+^ channels. The decay of intracellular Ca^2+^ concentration after termination of Ca^2+^ influx is determined by intracellular Ca^2+^ buffering properties and Ca^2+^ extrusion mechanisms.

Using this pre-pulse protocol (see Figure 8B), the effects of Cu^2+^-induced blockade of Ca^2+^ influx on I_KCa-v_ activation were evaluated. Figure 8A shows the time course of current inhibition produced by Cu^2+^ at concentrations of 0.3, 1, 3, and 10 µM. Cu^2+^ reduced I_KCa-v_ by 18 ± 3% at 0.3 µM (n = 5), 33 ± 3% at 1 µM (n = 5), 55 ± 2% at 3 µM (n = 6), and 70 ± 2% at 10 µM (n = 5), from an initial I_KCa-v_ amplitude of 1169 ± 74 nA. Figure 8B shows representative I_KCa-v_ current traces under control conditions (a) and after 2 min superfusion with Cu^2+^ (10 μM) (b). Inhibition of I_KCa-v_ was measured in each individual cell at the end of the 2 min superfusion period for each Cu^2+^ concentration, and the calculated IC_50_ value was 1.39 μM (Figure 8C).

**Figure 9:**
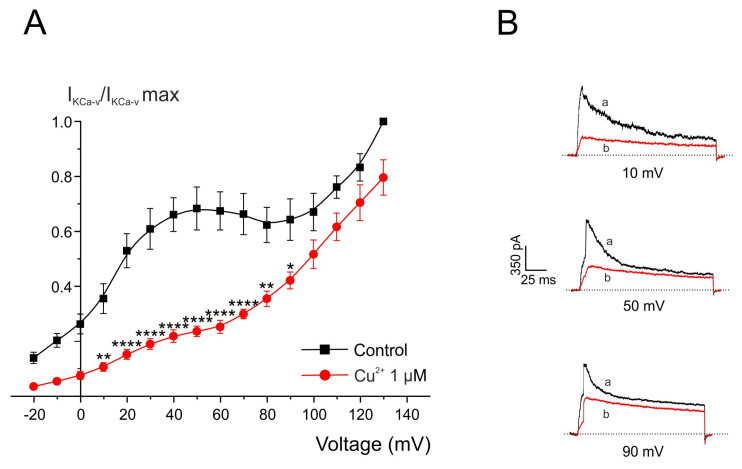
Voltage/K^+^-current curves obtained before and after perfusing with Cu^2+^. (**A**) Current–voltage (I–V) relationship of outward K^+^ currents recorded during test depolarizations before and after superfusion with Cu^2+^ (1 µM, 2 min). Note that the hump corresponding to the Ca^2+^/voltage-dependent K^+^ current is preferentially reduced after Cu^2+^ application. Data were normalized to the maximal control current for each cell and are expressed as mean ± SEM (n = 5 experiments). (**B**) Representative current traces recorded at the indicated test potentials under control conditions (a) and after superfusion with Cu^2+^ (1 µM, 2 min) (b). * *p* < 0.05, ** *p* < 0.01, and **** *p* < 0.0001 compared with control current at each potential (Wilcoxon matched-pairs signed-rank test).

Depolarizing steps that induce Ca^2+^ influx through voltage-dependent Ca^2+^ channels also activate Ca^2+^/voltage-dependent K^+^ channels, which can be identified by a characteristic hump in the current–voltage (I–V) relationship (Figure 9A). In this set of experiments, the effects of Cu^2+^ on the Ca^2+^-activated voltage-dependent potassium current (I_KCa-v_) were further examined.

Outward K^+^ currents were activated by 400 ms depolarizing pulses applied every 10 s from a holding potential of −80 mV in +10 mV increments, using 2.5 mM external Ca^2+^ as a function of membrane potential. Under control conditions, I_KCa-v_ showed a threshold of activation at approximately −20 mV, and the I–V relationship displayed a pronounced hump. This hump closely resembled the current–voltage relationship of the Ca^2+^ current in these cells, consistent with the Ca^2+^ dependence of this potassium current.

After 2 min application of Cu^2+^ (1 μM), a substantial reduction in peak I_KCa-v_ was observed. Following Cu^2+^ treatment, the residual current–voltage relationship exhibited a more linear profile, indicating a reduced contribution of Ca^2+^-activated K^+^ channels to the total outward current.

Figure 9B shows representative current traces recorded at +10 mV, +50 mV, and +90 mV under control conditions and after application of Cu^2+^ (1 μM). A marked reduction in I_KCa-v_ amplitude was observed after Cu^2+^ exposure, particularly between +10 mV and +90 mV, where the contribution of Ca^2+^/voltage-dependent K^+^ channels to the total outward K^+^ current is greater than at more depolarized potentials.

### 2.6. Effects of Cu^2+^ on the Cytosolic Calcium Transients

After observing that Cu^2+^ enhanced neurotransmitter release evoked by depolarizing stimuli with high extracellular K^+^ concentrations, while simultaneously inhibiting Ca^2+^ entry currents as well as I_Na_, I_K_, and I_KCa-v_, this apparent paradox led us to investigate whether Cu^2+^ could affect cytosolic calcium levels, which may modulate both neurotransmitter release and voltage-dependent calcium channels. Such an effect could help explain the increase in neurotransmission despite the inhibition of I_Ca_.

**Figure 10:**
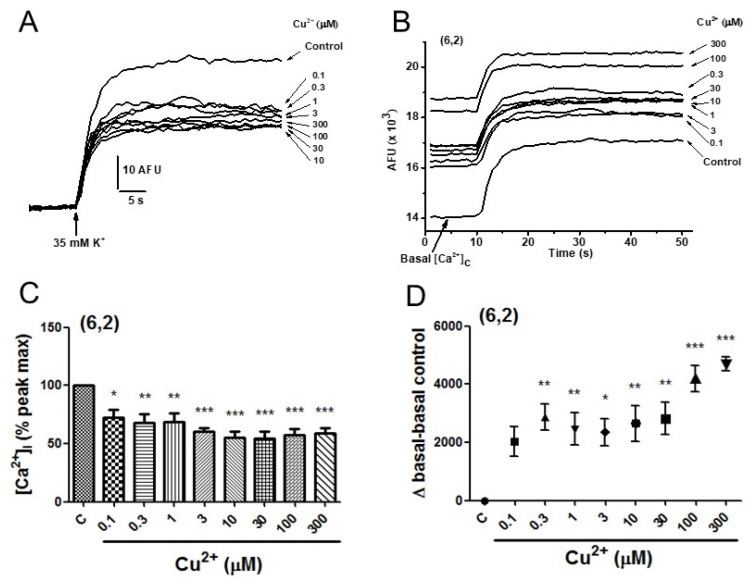
Effects of Cu^2+^ on basal and K^+^-evoked cytosolic Ca^2+^ signals in fluo-4-loaded BCCs. Cells seeded in black 96-well plates were exposed to increasing concentrations of Cu^2+^ 10 s before stimulation with a depolarizing pulse of 35 mM K^+^. (**A**) Family of cytosolic calcium concentration ([Ca^2+^]c) traces normalized to the initial baseline obtained under control conditions and in the presence of the indicated Cu^2+^ concentrations. Calibration bars indicate changes in arbitrary fluorescence units (AFU) versus time (s). (**B**) Representative [Ca^2+^]c signals elicited by 35 mM K^+^ expressed in absolute AFU (ordinate axis) versus time (abscissa axis), recorded under control conditions and after exposure of the cells to increasing Cu^2+^ concentrations. Note the increase in basal [Ca^2+^]c levels in the presence of Cu^2+^. Curves represent averages from 9 experiments performed in two different cell cultures. (**C**) Quantitative averaged data showing the net increase in [Ca^2+^]c elicited by 35 mM K^+^ in the absence (control) and presence of Cu^2+^ (indicated on the bottom horizontal line). Data were normalized to the peak control response and are expressed as mean ± SEM. (**D**) Net increases in basal fluorescence (Δ basal − basal control, AFU) induced by increasing Cu^2+^ concentrations prior to stimulation with 35 mM K^+^. Data in (**C**,**D**) are expressed as mean ± SEM of 6 experiments from two different cell cultures. * *p* < 0.05, ** *p* < 0.01, *** *p* < 0.001 compared with control.

To address this question, cytosolic Ca^2+^ mobilization was measured in populations of chromaffin cells stimulated with high K^+^ concentrations (35 mM). Cells were loaded with Fluo-4 AM and fluorescence signals were recorded (see Section 4). As shown in Figure 10, Cu^2+^ reduced the increase in cytosolic calcium concentration ([Ca^2+^]_c_) elicited by depolarizing pulses of 35 mM K^+^. However, Cu^2+^ markedly increased basal cytosolic calcium levels prior to stimulation.

Figure 10A shows a family of [Ca^2+^]_c_ traces normalized to the initial baseline under control conditions and in the presence of the indicated Cu^2+^ concentrations. Calibration bars represent changes in arbitrary fluorescence units (AFU) over time (s). Figure 10B shows representative [Ca^2+^]_c_ signals expressed in absolute AFU values as a function of time. These traces illustrate the increase in basal [Ca^2+^]_c_ induced by exposure to increasing Cu^2+^ concentrations before stimulation with 35 mM K^+^ pulses, as well as the subsequent Ca^2+^ elevations elicited by depolarization. Notably, the basal [Ca^2+^]_c_ level progressively increased with Cu^2+^ concentration in each trace. Curves represent averages from six experiments performed in two different cell cultures.

Quantitative averaged data for the net [Ca^2+^]_c_ increase elicited by 35 mM K^+^ are summarized in Figure 10C. Cu^2+^ produced a concentration-dependent inhibition of the K^+^-evoked calcium signal, ranging from 25.7 ± 6.3% inhibition at 1 μM to 45.9 ± 7.7% inhibition at 10–30 μM Cu^2+^. Finally, Figure 10D shows the net increases in basal fluorescence (after subtracting the control baseline) induced by increasing Cu^2+^ concentrations before the 35 mM K^+^ stimulus. Cu^2+^ significantly elevated basal [Ca^2+^]_c_ starting at 0.1 μM in a concentration-dependent manner, reaching values above 4000 AFU at higher Cu^2+^ concentrations.

**Figure 11:**
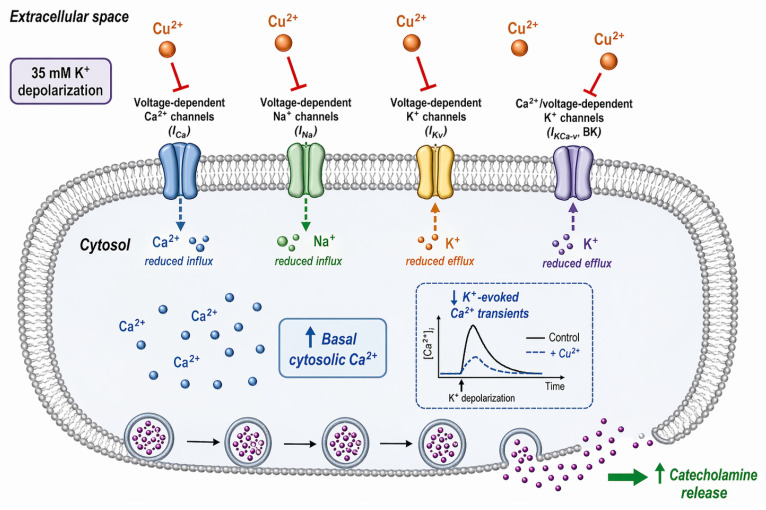
Proposed mechanism underlying the effects of Cu^2+^ on ionic currents, cytosolic Ca^2+^, and catecholamine release in bovine chromaffin cells. Acute Cu^2+^ exposure inhibits major voltage-dependent ionic currents, including Ca^2+^ currents (I_Ca_), Na^+^ currents (I_Na_), voltage-dependent K^+^ currents (I_Kv_), and Ca^2+^/voltage-dependent K^+^ currents (I_KCa-v_, BK). As a consequence, Cu^2+^ reduces Ca^2+^ influx and decreases the amplitude of K^+^-evoked cytosolic Ca^2+^ transients. At the same time, Cu^2+^ increases basal cytosolic Ca^2+^ levels. The combined effect of these actions may explain the paradoxical potentiation of catecholamine release observed in the presence of Cu^2+^, suggesting that disruption of intracellular Ca^2+^ homeostasis facilitates exocytosis despite the inhibition of ionic currents involved in cellular excitability.

Finally, Figure 11 summarizes the main findings of the present study and illustrates the mechanism proposed to explain the paradoxical effect of Cu^2+^ in bovine chromaffin cells. Acute exposure to Cu^2+^ inhibits the major voltage-dependent ionic currents involved in cellular excitability, including Ca^2+^ currents (I_Ca_), Na^+^ currents (I_Na_), voltage-dependent K^+^ currents (I_Kv_), and Ca^2+^/voltage-dependent K^+^ currents (I_KCa-v_, BK). In parallel, Cu^2+^ reduces the cytosolic Ca^2+^ transients evoked by depolarization with high extracellular K^+^, consistent with the inhibition of Ca^2+^ entry through voltage-dependent Ca^2+^ channels. However, Cu^2+^ also increases basal cytosolic Ca^2+^ levels. Taken together, these findings suggest that the enhancement of catecholamine release observed in the presence of Cu^2+^ is not due to an increase in depolarization-evoked Ca^2+^ influx, but rather to an alteration of intracellular Ca^2+^ homeostasis, whereby the elevation of basal cytosolic Ca^2+^ facilitates exocytosis despite the inhibition of inward ionic currents.

## 3. Discussion

In the present study, we analyzed the effects of acute copper (Cu^2+^) exposure on the excitability of bovine chromaffin cells by examining its effects on voltage-dependent calcium, sodium, and potassium currents, Ca^2+^-activated potassium currents, intracellular calcium dynamics, and neurotransmitter release. Our results show that Cu^2+^ produces multiple effects on chromaffin cells: (1) an increase in catecholamine release, (2) a gradual and partially reversible blockade of voltage-dependent Ca^2+^ currents, (3) a pronounced inhibition of voltage-dependent Na^+^ currents, (4) inhibition of voltage-dependent K^+^ conductance, (5) inhibition of Ca^2+^/voltage-dependent K^+^ currents, and (6) an increase in intracellular calcium levels.

Copper is an essential trace element that plays important roles in numerous biological and metabolic processes [12]. However, excessive or uncontrolled exposure to copper can have detrimental effects on human health [15]. Environmental copper levels have increased due to anthropogenic activities such as mining, industrial processes, and fossil fuel combustion, leading to increased human exposure [42]. Copper can enter the body through ingestion of contaminated water or inhalation of airborne particles [1], and copper compounds are widely used in agriculture and industry, further contributing to exposure [43]. Once in the body, copper can accumulate and has been associated with neurological disorders and neurodegenerative diseases such as Parkinson’s disease, Alzheimer’s disease, and amyotrophic lateral sclerosis [1]. One of the primary mechanisms proposed for copper neurotoxicity involves the generation of reactive oxygen species (ROS), leading to oxidative stress and neuronal damage [11].

In this study, we focused on the acute effects of Cu^2+^ on ionic currents responsible for cellular excitability and neurotransmitter release. We found that acute Cu^2+^ exposure stimulates evoked catecholamine release in a concentration-dependent manner. Calcium ions play a fundamental role in neurotransmitter release due to their influx through voltage-dependent calcium channels, mainly N- and P-type channels in neurons and L-type channels in neuroendocrine cells [44,45,46]. Our results show that I_Ca_ is blocked by Cu^2+^ in a time- and dose-dependent manner, with a reversible blockade and an IC_50_ of 45.7 µM. At the highest concentrations tested (300 µM), calcium influx was reduced by approximately 90%, indicating that multiple Ca^2+^ channel subtypes present in chromaffin cells are affected. The inhibition of I_Ca_ was more pronounced at negative potentials, and Cu^2+^ produced a slight shift in the I–V relationship toward more negative voltages.

Voltage-dependent Na^+^ channels are responsible for the depolarizing current that initiates action potentials in most excitable cells [47]. Our results show that Cu^2+^ blocks sodium currents in a time- and dose-dependent and reversible manner, with an IC_50_ of 17.00 µM. The highest concentration tested (30 µM) completely suppressed I_Na_, indicating a strong effect of Cu^2+^ on Na^+^ channel activity. However, Cu^2+^ did not significantly shift the I–V relationship or alter the kinetic parameters of sodium currents.

Potassium channels play a crucial role in repolarization of the action potential, regulation of resting membrane potential, and modulation of cellular excitability and firing patterns [48]. In chromaffin cells, potassium currents are mediated by both voltage-dependent K^+^ channels and Ca^2+^-activated K^+^ channels (BK channels) [49,50]. Our results show that Cu^2+^ inhibits voltage-dependent K^+^ currents with an IC_50_ of 11.25 µM without significant changes in the I–V relationship. Additionally, Cu^2+^ inhibited BK currents in a reversible and concentration-dependent manner, with an IC_50_ of 1.39 µM, indicating that Ca^2+^-activated K^+^ channels are particularly sensitive to Cu^2+^.

The mechanisms responsible for cytosolic calcium extrusion are essential for maintaining calcium homeostasis and include the Na^+^/Ca^2+^ exchanger [51], plasma membrane Ca^2+^-ATPase [52], sarco/endoplasmic reticulum Ca^2+^-ATPase (SERCA) [53], and mitochondrial calcium transport systems [54,55]. These mechanisms work together to maintain cytosolic calcium within a physiological range. The blockade of these mechanisms by Cu^2+^ could lead to accumulation of calcium in the cytosol, which in turn could result in excessive neurotransmitter release and disruption of synaptic signaling. Elevated cytosolic calcium may also induce mitochondrial production of reactive oxygen species, contributing to oxidative stress and neuronal dysfunction.

One of the most important findings of this study is the apparent paradox that Cu^2+^ enhances neurotransmitter release while simultaneously inhibiting voltage-dependent Ca^2+^ currents. Measurements of cytosolic Ca^2+^ provide a possible explanation for this observation. Cu^2+^ significantly increased basal cytosolic Ca^2+^ levels while reducing the Ca^2+^ transients evoked by depolarization with high K^+^. This suggests that Cu^2+^ disrupts intracellular calcium homeostasis, possibly by inhibiting calcium extrusion mechanisms or by mobilizing calcium from intracellular stores such as the endoplasmic reticulum or mitochondria. The increase in basal cytosolic Ca^2+^ could facilitate vesicle priming and fusion, thereby enhancing neurotransmitter release despite reduced Ca^2+^ influx through voltage-dependent calcium channels.

Additionally, Cu^2+^ has been reported to inhibit Na^+^/K^+^-ATPase activity, which could increase intracellular Na^+^ concentration and reverse the operation of the Na^+^/Ca^2+^ exchanger, leading to increased intracellular Ca^2+^ levels [56]. Heavy metals, including Cu^2+^, also inhibit plasma membrane Ca^2+^-ATPase activity at micromolar concentrations [57,58]. Since calcium transport ATPases and copper transport ATPases belong to the P-type ATPase family and share similar catalytic mechanisms, Cu^2+^ may interfere with Ca^2+^ transport processes when present in the cytosol [59,60]. Furthermore, Cu^2+^ may affect mitochondrial function and the mitochondrial calcium uniporter, further contributing to alterations in intracellular calcium regulation [61].

An additional point that deserves consideration is the coordinated role of inward and outward ionic currents in shaping catecholamine secretion. Recent reviews emphasize that stimulus–secretion coupling in chromaffin cells is not determined exclusively by the magnitude of Ca^2+^ entry, but by the dynamic interaction between depolarizing and repolarizing conductances that define action potential waveform, firing frequency, and intracellular Ca^2+^ handling [38,62]. At the onset of stimulation, inward cationic currents through nicotinic receptors, together with voltage-dependent Na^+^ currents, provide the depolarizing drive required to recruit high-voltage-activated Ca^2+^ channels [63]. Ca^2+^ influx through these channels is the immediate trigger for vesicle fusion and catecholamine release. However, recent evidence indicates that the final secretory response also depends on the contribution of intracellular Ca^2+^ stores and mitochondrial Ca^2+^ buffering, indicating that exocytosis reflects integrated Ca^2+^ homeostasis rather than Ca^2+^ influx alone. In parallel, outward K^+^ currents, including voltage-dependent K^+^ currents and Ca^2+^-activated K^+^ currents, are critical for action potential repolarization, spike duration, and interspike interval, and therefore strongly influence both the amount and temporal profile of Ca^2+^ entry [38]. From this perspective, inhibition of outward K^+^ conductances may prolong depolarization and transiently favor exocytosis, whereas inhibition of inward Na^+^ and Ca^2+^ currents would be expected to reduce cellular excitability and secretory capacity. Thus, the final effect of Cu^2+^ on catecholamine release is likely to result from the balance between these opposing actions on membrane excitability and from its additional ability to alter basal cytosolic Ca^2+^ levels.

Overall, our findings indicate that Cu^2+^ exerts a dual effect on neuronal function by inhibiting ionic currents required for action potential generation and propagation while simultaneously enhancing neurotransmitter release through disruption of intracellular calcium homeostasis. The paradoxical increase in exocytosis despite inhibition of Ca^2+^ entry currents can be explained by an elevation of basal cytosolic Ca^2+^ levels and altered calcium handling mechanisms.

Another mechanism that may contribute to the effects of Cu^2+^ is its behavior as a labile extracellular cation capable of modifying the ionic microenvironment at the outer membrane surface. Current reviews indicate that weakly bound copper pools can be released during neuronal activity and transiently accumulate in restricted extracellular compartments, where they modulate neuronal excitability and Ca^2+^ homeostasis [64,65,66]. In this context, extracellular Cu^2+^ may alter the local electrostatic environment near the membrane by screening negatively charged surface groups and by competing with other cations for external binding sites, thereby modifying channel gating and voltage dependence [65].

In addition to these surface charge effects, Cu^2+^ can also act through direct coordination to membrane proteins, frequently involving extracellular amino acid residues able to bind transition metals. In agreement with this idea, direct extracellular Cu^2+^-binding sites have been identified in BK and Shaker K^+^ channels, where Cu^2+^ produces rapid and reversible inhibition [67], and extracellular Cu^2+^ has also been shown to inhibit ENaC through binding to multiple extracellular sites [68]. Moreover, recent reviews emphasize that copper interacts not only with ion channels themselves but also with copper-binding and copper-transporting membrane proteins, including APP, PrP, CTR1, and ATP7A/B, which participate in copper buffering, uptake, reduction, and redistribution at the cell surface [65,66].

Therefore, the effects of Cu^2+^ on catecholamine secretion may reflect both a disturbance of the extracellular cationic microenvironment and direct interactions with channels and membrane proteins that regulate ion fluxes and Ca^2+^ signaling.

In summary, acute copper exposure significantly alters cellular excitability and calcium homeostasis in chromaffin cells. Cu^2+^ inhibits calcium, sodium, and potassium currents involved in the initiation, propagation, and termination of action potentials, while simultaneously increasing intracellular calcium levels and enhancing neurotransmitter release. These findings suggest that copper-induced dysregulation of calcium homeostasis may play a key role in the neurotoxic effects of copper and its impact on synaptic transmission and neuronal function.

Several limitations of the present study should be acknowledged. First, the experiments were performed in primary bovine chromaffin cells, a well-established neuroendocrine model for studying cellular excitability and catecholamine secretion, but one that does not fully reproduce the complexity of synaptic transmission in intact neuronal circuits. Therefore, caution is needed when extrapolating these findings directly to central neurons or to in vivo conditions. Second, the study focused on the acute effects of Cu^2+^ exposure. Consequently, the results do not address the possible consequences of chronic copper accumulation, adaptive cellular responses, or long-term toxic effects, which may involve additional mechanisms such as oxidative stress, altered gene expression, mitochondrial dysfunction, or progressive impairment of membrane transport systems. Third, although our data show that Cu^2+^ increases basal cytosolic Ca^2+^ while inhibiting voltage-dependent ionic currents, the precise intracellular sources of this Ca^2+^ rise and the molecular targets responsible for the disruption of calcium homeostasis were not directly identified. In addition, the contribution of copper interactions with extracellular membrane surface charges, ion-channel binding sites, or copper-transporting proteins was not specifically examined in the present work.

Despite these limitations, the present findings may have several relevant applications. From a mechanistic perspective, this study provides a useful framework for understanding how acute copper exposure uncouples membrane excitability from secretory output by simultaneously inhibiting major ionic currents and increasing basal cytosolic Ca^2+^ levels. This may be of interest for future studies on metal-induced dysregulation of neurosecretion and on the cellular basis of copper neurotoxicity. In addition, bovine chromaffin cells may serve as an experimental platform for testing pharmacological strategies aimed at preventing copper-induced alterations in ion-channel function and calcium homeostasis. More broadly, these results may help guide future investigations into the effects of copper imbalance in excitable cells, including neuroendocrine cells and neurons, and may contribute to a better understanding of the cellular events associated with disorders of copper homeostasis.

## 4. Materials and Methods

### 4.1. Isolation and Culture of Bovine Chromaffin Cells

All procedures involving animals were conducted in accordance with the guidelines of the National Council on Animal Care and the European Communities Council Directive (86/609/EEC) and were approved by the Animal Care Committee of the Universidad Autónoma de Madrid (ES280790000092) on 18 November 2021.

Chromaffin cells, like sympathetic neurons, originate from the neural crest. Due to their neuron-like electrical excitability and their ability to synthesize, store, and release adrenaline and noradrenaline, chromaffin cells are widely used as a cellular model for studying cellular excitability and neurotransmitter release [32,33,35,36,37,38].

In accordance with bioethical animal welfare practices and European regulations (EC No. 1099/2009), Spanish legislation requires procedures that minimize animal suffering during slaughter. Adrenal glands were obtained from a local slaughterhouse under veterinary supervision. Animals were stunned using a captive bolt pistol prior to slaughter, and bleeding was initiated immediately after stunning.

Bovine chromaffin cells were isolated from the adrenal medulla by enzymatic digestion with collagenase. A total of 24 adrenal glands from 12 animals were used to obtain 12 primary cell cultures. For each primary culture, two adrenal glands were pooled before cell plating. Figure legends indicate the number of cells and the number of cultures used in each experimental group.

Cells were suspended in Dulbecco’s Modified Eagle’s Medium (DMEM, Thermo Fisher, Waltham, MA, USA) supplemented with 5% fetal bovine serum, 50 IU/mL penicillin, and 50 μg/mL streptomycin. To prevent excessive fibroblast proliferation, proliferation inhibitors were added to the culture medium (10 μM cytosine arabinoside, 10 μM fluorodeoxyuridine, and 10 μM leucine methyl ester).

For amperometric secretion experiments, cells were plated on 10 cm diameter Petri dishes at a density of 5 × 10^6^ cells in 10 mL of DMEM. For patch-clamp experiments, cells were plated at low density on 1 cm diameter glass coverslips (5 × 10^4^ cells per coverslip). For intracellular calcium measurements, cells were plated at a density of 2 × 10^5^ cells per well in 96-well plates.

Cell cultures were maintained in an incubator at 37 °C in a humidified atmosphere containing 5% CO_2_, and cells were used between 1 and 4 days after plating.

### 4.2. On-Line Measurement of Neurotransmitter Release

The electrochemical method used to measure exocytosis is based on the ability of catecholamines to undergo oxidation–reduction reactions at the surface of a carbon fiber electrode [69]. Bovine chromaffin cells were carefully scraped from the bottom of Petri dishes using a rubber spatula and centrifuged at 120 *g* for 10 min. The resulting cell pellet was resuspended in 200 μL of Krebs–HEPES (Sigma-Aldrich/Merck KGaA, Darmstadt, Germany) solution at pH 7.4 containing (in mM): 144 NaCl, 5.9 KCl, 1.2 MgCl_2_, 11 glucose, 10 HEPES, and 2 CaCl_2_.

Cells were then placed in a microchamber (100 μL volume) and superfused at 37 °C with Krebs–HEPES solution at a flow rate of 2 mL/min. Under these conditions, the perfusion fluid leaving the microchamber reached an electrochemical detector (model CH-9100, Metrohm AG, Herisau, Switzerland) equipped with a carbon fiber microelectrode positioned at the outlet of the chamber. Catecholamines released from the cells were oxidized at a potential of 650 mV, and the resulting oxidation current was recorded at a sampling frequency of 2 Hz to monitor total catecholamine secretion.

Catecholamine release was stimulated by applying a Krebs–HEPES solution containing 35 mM KCl, with isosmotic reduction in NaCl (35K^+^ solution), in 5 s pulses delivered at 1 min intervals at 37 °C. After each stimulation, catecholamine release was measured in real time by amperometry [70].

The total charge (Q) released during secretion was calculated by integrating the current over time according to Faraday’s law, expressed as: Q = nNF where n is the number of electrons transferred in the redox reaction (n = 2 for catecholamines), N is the number of neurotransmitter molecules detected, and F is Faraday’s constant. Solutions were rapidly exchanged using electronically controlled valves operated by a computer.

### 4.3. Electrophysiological Recording and Data Analysis

Voltage-clamp recordings were performed using the whole-cell configuration of the patch-clamp technique. Patch pipettes were fabricated from thin-wall fire-polished borosilicate glass (Kimax 51, Witz Scientific, Holland, OH, USA), resulting in a final series resistance of 5–7 MΩ when filled with standard intracellular solution. Pipettes were mounted on the headstage of an EPC-9 patch-clamp amplifier and recordings were controlled using PatchMaster software version 2.92 (HEKA Electronic, Lambrecht/Pfalz, Germany). Recordings were initiated when access resistance decreased below 20 MΩ. Series resistance was compensated by 80% and continuously monitored throughout the experiments.

For recordings of Ca^2+^ (I_Ca), Na^+^ (I_Na), and K^+^ (I_K) currents, as well as for current-clamp experiments, data were acquired at sampling frequencies between 5 and 10 kHz and filtered at 1–2 kHz. Recording traces showing leak currents greater than 100 pA in voltage-clamp mode or series resistance greater than 20 MΩ were discarded.

During seal formation, cells were continuously perfused with Tyrode solution containing (in mM): 137 NaCl, 5 KCl, 1 MgCl_2_, 2 CaCl_2_, and 10 HEPES/NaOH (pH 7.4). After membrane rupture and establishment of the whole-cell configuration, cells were superfused with modified Tyrode solutions depending on the current being recorded. Tyrode solution containing nominally 0 mM Ca^2+^ was used to record I_Na_; Tyrode solution containing 2.5 mM Ca^2+^ was used to record I_KCa-v_ and I_Kv_; and Tyrode solution containing 10 mM Ca^2+^ plus 1 µM tetrodotoxin (TTX) was used to record I_Ca_ in order to block sodium currents (see Section 2 for specific voltage protocols).

For I_Na_ and I_Ca_ recordings, cells were dialyzed with an intracellular solution containing (in mM): 10 NaCl, 100 CsCl, 14 EGTA, 20 TEA-Cl, 5 Mg-ATP, 0.3 Na-GTP, and 20 HEPES/CsOH (pH 7.4 adjusted with CsOH). For I_K_ recordings and current-clamp experiments, CsCl and TEA-Cl were replaced with KCl, and pH was adjusted to 7.4 with KOH.

External solutions were exchanged using a pump-driven perfusion system at a flow rate of 2 mL/min, allowing complete solution exchange within approximately 20 s.

### 4.4. Monitoring of Cytosolic Calcium Levels

To monitor changes in cytosolic calcium concentration ([Ca^2+^]_c_), cells were plated at a density of 2 × 10^5^ cells per well in black 96-well plates, and experiments were performed 48 h after plating. Cells were loaded with Krebs–HEPES solution containing (in mM): 144 NaCl, 5.9 KCl, 1.2 MgCl_2_, 11 glucose, 10 HEPES, and 2 CaCl_2_ (pH 7.4 adjusted with NaOH), supplemented with 10 µM Fluo-4 AM and 0.2% pluronic acid. Cells were incubated for 45 min at 37 °C in the dark.

After incubation, cells were washed twice with Krebs–HEPES solution at room temperature in the dark. Changes in fluorescence were measured using a fluorescence plate reader (Fluostar, BMG Labtech, Offenburg, Germany) with excitation at 485 nm and emission at 520 nm. Basal fluorescence levels were recorded before addition of the stimulation solution (35K^+^) using an automatic dispenser. Following stimulation, fluorescence changes were recorded for 60 s.

To normalize Fluo-4 signals, responses from each well were calibrated by measuring maximum and minimum fluorescence values. At the end of each experiment, 5% Triton X-100 was added to obtain maximal fluorescence (F_max), followed by addition of 2 mM MnCl_2_ to obtain minimal fluorescence (F_min). Data were expressed as a percentage of the F_max–F_min range.

### 4.5. Chemicals

CuCl and the salts used for preparation of saline solutions were obtained from Merck (Madrid, Spain). Collagenase type I was purchased from Roche Laboratories (Madrid, Spain). Dulbecco’s Modified Eagle’s Medium (DMEM), fetal bovine serum (fraction V), and penicillin–streptomycin were obtained from Gibco (Madrid, Spain). Pluronic acid was purchased from Sigma-Aldrich/Merck KGaA, Germany, and Fluo-4 AM was obtained from Invitrogen (Thermo Fisher Scientific, Waltham, MA, USA). All other chemicals and reagents were purchased from Sigma-Aldrich/Merck KGaA, Germany and Panreac Chemical, Barcelona, Spain.

### 4.6. Statistical Analysis

Data are expressed as mean ± SEM of the number of cells studied (n), obtained from at least three independent cell cultures. Data were first evaluated for normality using the D’Agostino–Pearson omnibus normality test. Since some parameters followed a normal distribution whereas others did not, statistical comparisons were performed using the Wilcoxon matched-pairs signed-rank test in all cases. Statistical significance was established at *p* < 0.05. Concentration–response curves and IC_50_ values were obtained by fitting the data to a sigmoidal Hill equation: y = (V_max · x^n^)/(k^n^ + x^n^). All statistical analyses and curve fittings were performed using GraphPad Prism software (version 8.01).

## 5. Conclusions

In the present study, we investigated the effects of acute Cu^2+^ exposure on ionic currents, catecholamine release, and cytosolic Ca^2+^ dynamics in bovine chromaffin cells. Our results show that Cu^2+^ exerts a dual action on these cells. On the one hand, Cu^2+^ increased depolarization-evoked catecholamine release and elevated basal cytosolic Ca^2+^ levels. On the other hand, Cu^2+^ inhibited voltage-dependent Ca^2+^, Na^+^, and K^+^ currents, as well as Ca^2+^/voltage-dependent K^+^ currents, in a concentration-dependent and partially reversible manner.

These findings demonstrate that acute Cu^2+^ exposure disrupts the ionic mechanisms that control cellular excitability and alters intracellular Ca^2+^ homeostasis in bovine chromaffin cells. The enhancement of catecholamine release despite the inhibition of inward Ca^2+^ current is consistent with the increase in basal cytosolic Ca^2+^ levels observed in the presence of Cu^2+^.

Overall, these findings indicate that the neurotoxic effects of Cu^2+^ may be associated with alterations in ionic currents that regulate cellular excitability and neurotransmitter release, as well as disruption of intracellular calcium homeostasis. These alterations may contribute to the effects of copper on neuronal function and synaptic transmission.

## Data Availability

The original contributions presented in this study are included in the article. Further inquiries can be directed to the corresponding author.
